# Chronische Rhinosinusitis mit nasalen Polypen – Verlängerung der Dupilumab-Therapieintervalle

**DOI:** 10.1007/s00106-024-01487-y

**Published:** 2024-05-18

**Authors:** H. M. Appel, R. Lochbaum, T. K. Hoffmann, J. Hahn

**Affiliations:** Universitätsklinik für Hals‑, Nasen‑, Ohren-Heilkunde, Kopf- und Halschirurgie, Universitätsklinikum Ulm, Frauensteige 12, 89075 Ulm, Deutschland

**Keywords:** Rhinitis, Nasenkrankheiten, Biologika, Monoklonale humanisierte Antikörper, Asthma, Rhinitis, Nose diseases, Biologic drugs, Monoclonal humanized antibodies, Asthma

## Abstract

**Hintergrund:**

Eine schwere, nicht kontrollierte chronische Rhinosinusitis mit nasalen Polypen (CRSwNP) kann sich unter Dupilumab 300 mg 2‑wöchentlich komplett zurückbilden. Spätestens dann folgt die Frage der Patienten nach einer möglichen Therapiedeeskalation. Eine Beendigung der Antikörpertherapie nach 24 Wochen führte zum Rezidiv, hingegen blieb unter reduzierter Dupilumab-Dosis durch Streckung des Intervalls auf 4 Wochen die Kontrolle erhalten. Eine vom Zulassungstext abweichende Verlängerung der Therapieintervalle wird jedoch aktuell nicht empfohlen.

**Methoden:**

Es erfolgte eine retrospektive Untersuchung des Verlaufs von 29 Patienten mit schwerer CRSwNP, mit Typ-2-Inflammation assoziierten Komorbiditäten und bestehender Indikation zur Biologikatherapie. Nach Rückbildung der CRSwNP und der Beschwerden unter Dupilumab 300 mg 2‑wöchentlich war das Applikationsintervall individuell zunächst auf 4 Wochen, danach ggf. auf 6 Wochen verlängert worden. Erfasst wurden u. a. die Lebensqualität (Sinonasal Outcome Test, SNOT-22), der nasale Polypenscore (NPS) und das Riechvermögen (Sniffinʼ Sticks, Fa. Burghart Messtechnik, Holm, Deutschland).

**Ergebnisse:**

Alle Patienten zeigten innerhalb der ersten 3 Monate ein sehr gutes Therapieansprechen. Eine Verlängerung des Dupilumab-Intervalls auf 4 Wochen erfolgte nach 7–31 Monaten (median 13 Monate), auf 6 Wochen (*n* = 9) nach 17–35 Monaten (median 23 Monate). Im Verlauf traten bei keinem Patienten ein Rezidiv, eine Verschlechterung der Lebensqualität oder des Riechens auf.

**Schlussfolgerung:**

Eine Verlängerung der Dupilumab-Injektionsintervalle auf 4 evtl. auch 6 Wochen ist individuell nach weitestgehender Rückbildung der Polypen und Beschwerden ohne klinische Verschlechterung möglich. Weitere Studien zur Deeskalation bzw. Beendigung der Biologikatherapie bei erzielter CRSwNP-Kontrolle sind unabdingbar.

## Abstract (en)

**Background:**

In patients with severe, uncontrolled chronic rhinosinusitis with nasal polyps (CRSwNP), dupilumab 300 mg every 2 weeks can completely resolve nasal polys, sinus disease, and symptoms. In this case, patients ask for de-escalation. Although trials have demonstrated recurrence after stopping the biologic at 24 weeks, reducing the dose of dupilumab to once every 4 weeks did not result in deterioration of control. An extension of the treatment intervals would, however, diverge from the approval text, and is currently not recommended.

**Methods:**

The course of 29 patients with severe CRSwNP, type‑2 inflammation-associated comorbidities, and an indication for biologic was retrospectively analyzed. After resolution of CRSwNP and symptoms under biweekly dupilumab 300 mg, the dupilumab interval had been prolonged individually, initially up to 4 weeks, thereafter up to 6 weeks, if applicable. Control was assessed via quality of life (22-item sinonasal outcome test, SNOT-22), nasal polyp score, and smell identification test (Sniffinʼ Sticks; Burghart Messtechnik, Holm, Germany).

**Results:**

All patients showed an excellent improvement within the first 3 months. The dupilumab application interval was extended to 4 weeks after 7–31 months (median 13 months) and to 6 weeks (*n* = 9) after 17–35 months (median 23 months). No recurrent polyps or symptoms were subsequently observed.

**Conclusion:**

In case of maximal regression of polyps and discomfort, extension of dupilumab injection intervals to 4 and potentially 6 weeks is possible without clinical worsening. Further studies on de-escalation or termination of biologic treatment when CRSwNP control is achieved are essential.

## Empfehlungen zur Biologikatherapie

Dupilumab (Anti-Interleukin[IL]-4Rα) ist seit Oktober 2019 als Add-on-Therapie mit intranasalen Kortikosteroiden zur Behandlung von Erwachsenen mit schwerer CRSwNP, die mit systemischen Kortikosteroiden und/oder chirurgischem Eingriff nicht ausreichend kontrolliert werden kann, zugelassen. Die entsprechende Applikation beträgt 300 mg im 2‑wöchentlichen Intervall. Eine schwere, Typ-2-Inflammation-assoziierte CRSwNP kann sich hierunter komplett zurückbilden. In diesem Fall fragen Patienten bei Beschwerdefreiheit meist nach einer möglichen Dosisreduktion oder Beendigung der Therapie.

Das EUFOREA (The European Forum for Research and Education in Allergy and Airway Diseases) Expert Board gibt jährlich aktuelle Empfehlungen zur Biologikatherapie bei schwerer CRSwNP, zur Diagnostik, Biologikaindikation, Entscheidungsfindung, zum Monitoring, zur Therapieevaluation und zum Therapiemanagement [2, 5, 6] sowie zur Definition von CRSwNP-Krankheitsaktivität, -Kontrolle, -Remission und -Therapiezielen [6]. Im Jahr 2021 wurde von einzelnen Autoren diskutiert, dass nach 3–5 Jahren Follow up unter optimalen Bedingungen (endoskopisch keine nachweisbaren Polypen mehr, keine oder geringe Symptome) eine Beendigung der Biologikatherapie erwogen werden kann, intranasale Kortikosteroide (INCS) weiter appliziert und die Patienten engmaschig (3 Monate) kontrolliert werden sollten [2, corrigenda]. Eine Beendigung der Biologikatherapie bei kompletter Rückbildung der Polypen und entsprechender Symptome im Sinne einer CRSwNP-Kontrolle [6] wird jedoch aufgrund fehlender evidenzbasierter Daten von EUFOREA nicht empfohlen [2, 5]. Im EPOS- (European Position Paper on Rhinosinusitis and Nasal Polyps)/EUFOREA-Update 2023 werden Untersuchungen zur Verlängerung der Biologikatherapieintervalle und zur Beendigung der Biologikatherapie diskutiert. Dabei wird die Notwendigkeit, ein Ende des Therapiezeitplans der Biologika für Patienten mit CRSwNP unter Kontrolle festzulegen, betont [5]. Asthmastudien zur Beendigung der Therapie mit Omalizumab und Mepolizumab ergaben, dass für geeignete Patienten die Beendigung der Biologikatherapie eine praktikable Strategie darstellen könnte. Aufgrund fehlender evidenzbasierter Ergebnisse von Patienten mit CRSwNP können hierzu derzeit noch keine eindeutigen Richtlinien gegeben werden [5]. Die Zulassungsstudien zeigten, dass nach 24 Wochen Dupilumab 300 mg 2‑wöchentlich eine Beendigung der Therapie zum Rezidiv führt, hingegen resultierte durch Verlängerung der Therapieintervalle auf 4 Wochen keine Verschlechterung des nasalen Polypenscores (NPS) bzw. der nasalen Obstruktion [1]. Im Fall einer Kontrolle der CRSwNP wäre eine Verlängerung der Biologikaintervalle medizinisch möglich, wird aber wegen der nicht zugelassenen Applikationsintervalle, daher potenziellem Off-Label-Use [7], nicht empfohlen [10]. Für das Asthma und die atopische Dermatitis wurde gezeigt, dass eine gute Kontrolle unter der Biologikatherapie mit verlängerten Therapieintervallen erhalten werden kann [3, 4, 11]. Hier stellt sich die Frage, ob die Kontrolle der CRSwNP im Sinne einer weitestgehenden Rückbildung der Polypen und Beschwerden unter Dupilumab erhalten werden kann, wenn die Dupilumab-Intervalle personalisiert auf 4 Wochen, ggf. weiter auf 6 Wochen extendiert werden.

## Methoden

Patienten mit CRSwNP mit Biologikatherapie wurden in der klinikeigenen elektronischen Patientenakte in einer Sprechstundenliste gesondert erfasst. Es erfolgte eine retrospektive Auswertung vorhandener Daten zum Verlauf von 29 Patienten (7 w, 22 m), 30–77 Jahre (Durchschnitt: 52 Jahre) mit schwerer CRSwNP und gegebener Indikation zur Biologikatherapie [5, 9], die Dupilumab 300 mg als Add-on zu INCS erhielten. War unter dem 2‑wöchigem Applikationsintervall eine weitestgehende Rückbildung der Polypen (NPS ≤ 1 einseitig), der sinunasalen Symptome mit entsprechender Besserung der Lebensqualität sowie auch Besserung der Symptome der unteren Atemwege erzielt worden und bestand der Patientenwunsch nach einer Therapiedeeskalation, war ein Antrag auf Kostenübernahme bei Verlängerung der Therapieintervalle gestellt worden. Nach Bewilligung durch den Versicherungsträger war das Injektionsintervall von Dupilumab 300 mg auf 4 Wochen verlängert worden. Bei darunter fortbestehender Kontrolle und Beschwerdefreiheit war eine weitere Intervallverlängerung nach frühestens 6 Monaten auf 6 Wochen sowie ggf. nach weiteren 6 Monaten auf 8 Wochen erfolgt. Beurteilt wurden neben der Anamnese die Lebensqualität, erfasst mit dem Sinonasal Outcome Test, SNOT-22, der endoskopisch erhobene nasale Polypenscore (NPS 0–8, einseitig 0–4) nach P. Gevaert et al. [8] sowie das Riechvermögen mit Sniffinʼ Sticks (Fa. Burghart Messtechnik, Holm, Deutschland). Die im Rahmen der Sprechstunde prätherapeutisch und i. d. R. in 3–6-monatigen Abständen dokumentierten Befunde wurden ausgewertet, eine Beurteilung der verlängerten Dupilumab-Applikationsintervalle erfolgte frühestens nach 6 Monaten.

## Ergebnisse

Alle Patienten hatten vor Einleitung der Dupilumab-Therapie überwiegend mehrfach operative Sanierungen der Nasennebenhöhlen (NNH), durchschnittlich 3 (1 ≤ *n* ≤ 7) und litten unter nachgewiesenen Typ-2-Inflammation-assoziierten Komorbiditäten, 27 unter einem Asthma bronchiale, therapiert mit inhalativen Kortikosteroiden und β2-Mimetika, 17 unter einer Intoleranz gegenüber nichtsteroidalen Antirheumatika (NSAID; „NSAID-exacerbated respiratory disease“, NERD) und 10 unter einer allergischen Rhinitis (Tab. 1).

**Tab. 1:** Patientencharakteristika vor Dupilumab hinsichtlich Anzahl der NNH-Sanierungen, des NPS (in Klammern Patientenzahl), Asthma, NSAID-Intoleranz (NERD) und allergischer Rhinitis

Anzahl der Patienten	NNH-Op.	NPS	Asthma	NERD	Allergische Rhinitis
6	1	5 (3), 6 (3)	6	2	1
9	2	5 (8), 6 (1)	9	4	1
6	3	5 (4), 6 (2)	5	6	6
5	4	4 (2), 5 (2)	5	3	2
2	5	5	–	1	1
1	7	5 (1), 6 (1)	2	1	–

*NERD* NSAID-Intoleranz („NSAID-exacerbated respiratory disease“); *NNH* Nasennebenhöhlen; *NPS* nasaler Polypenscore; *NSAID* nichtsteroidale Antirheumatika („nonsteroidal anti-inflammatory drugs“)

**Abb. 1:**
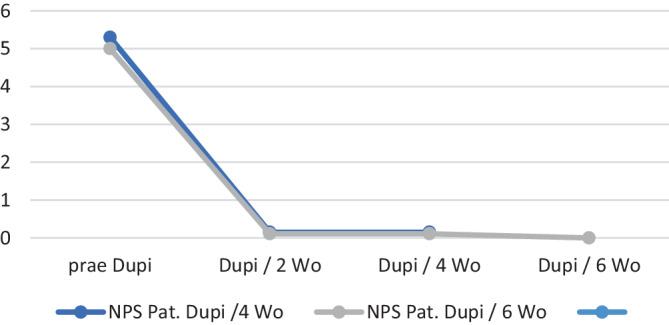
Nasaler Polypenscore (*NPS*; Mittelwert) vor Anwendung von Dupilumab (*prae Dupi*), unter 300 mg/2 Wochen sowie unter den auf 4 und 6 Wochen verlängerten Therapieintervallen; 20 Patienten (*blau*) mit Intervallverlängerung auf 4 Wochen, 9 Patienten (*grau*) mit Intervallverlängerung auf 6 Wochen

Dupilumab als Add-on erzielte bei allen Patienten innerhalb der ersten 3 Monate eine deutliche Rückbildung der Symptome und Polypen (Abnahme des NPS ≥ 2), einhergehend mit einer Verbesserung der Lebensqualität. Ebenso beschrieben die Patienten im Verlauf eine Besserung des Asthmas mit Reduktion der Medikation sowie eine Besserung der allergischen Rhinitis. Bei systemischen Kortikosteroiden konnte unter Dupilumab ein Ausschleichen erfolgen, eine erneute Gabe war in keinem Fall erforderlich. Nach weitestgehender Rückbildung der Polypen und Symptome im Sinne einer kompletten Kontrolle der CRSwNP sowie deutlicher Besserung der Lebensqualität und Komorbiditäten erfolgte die Verlängerung des Dupilumab-Intervalls auf 4 Wochen nach 7–31 Monaten (Median: 13 Monate), bei Patienten mit 3 oder mehr NNH-Operationen (*n* = 15) im Median nach 17 Monaten. Bei 9 Patienten wurde das Intervall weiter verlängert auf 6 Wochen im Median nach 23 Monaten (17–35 Monate), einer dieser Patienten extendierte das Dupilumab-Intervall nach 27 Monaten auf 8 Wochen. Im Verlauf traten bei keinem Patienten erneut Polypen, sinunasale Symptome oder eine Verschlechterung der Lebensqualität auf. Anamnestisch verschlechterte sich bei keinem Patienten das Asthma unter den verlängerten Therapieintervallen; regelmäßige, mindestens halbjährliche Lungenfunktionsprüfungen und lungenfachärztliche Kontrollen wurden durchgeführt. Der NPS betrug durchschnittlich vor Anwendung von Dupilumab 5,21 (4–6; SD 0,56), unter Dupilumab 300 mg/2 Wochen vor Verlängerung des Applikationsintervalls 0,14 (0–2; SD 0,46), unter Dupilumab 300 mg/4 Wochen ebenfalls 0,14 (0–2; SD 0,44) und unter Dupilumab 300 mg/6 Wochen 0,00 (Abb. 1).

**Abb. 2:**
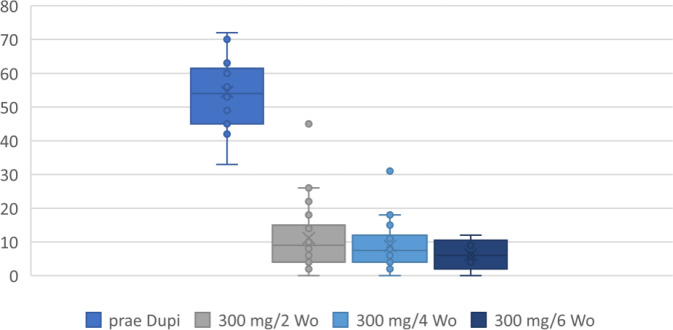
Sinonasal Outcome Test (SNOT-22) vor Dupilumab (*prae Dupi*), unter 300 mg/2 Wochen sowie unter den auf 4 und 6 Wochen verlängerten Therapieintervallen

Vor Dupilumab-Anwendung bestand eine erhebliche Einschränkung der Lebensqualität, im SNOT-22 mit durchschnittlich 53 Punkten (33–72; Standardabweichung, SD: 10,10) erfasst, die sich unter der Therapie hervorragend besserte. Durchschnittlich wurden im SNOT-22 nach der 2‑wöchigen Applikation vor Umstellung auf 300 mg/4 Wochen 11 Punkte (2–45; SD 10,14), unter 300 mg/4 Wochen 8 Punkte (2–31; SD 8,33) sowie unter 300 mg/6 Wochen 6 Punkte (0–12; SD 4,0) angegeben (Abb. 2).

**Abb. 3:**
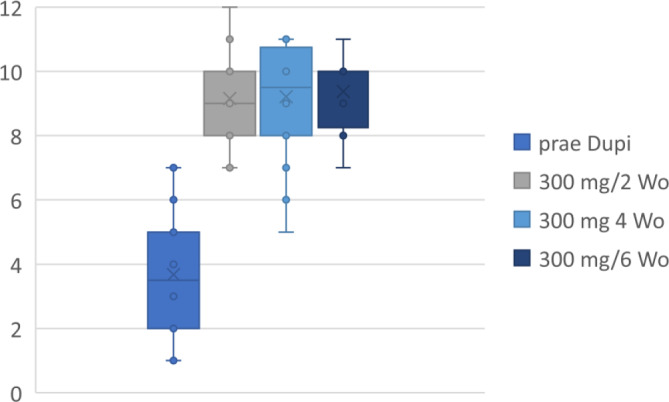
Erfassung des Riechvermögens mit Sniffinʼ Sticks (Fa. Burghart Messtechnik, Holm, Deutschland) vor Dupilumab (*prae Dupi*), unter 300 mg/2 Wochen, 300 mg/4 Wochen und 300 mg/6 Wochen

Vor der Anwendung von Dupilumab litten die Patienten überwiegend unter einer Anosmie, durchschnittlich wurden 3,6/12 Sniffinʼ Sticks (1–7; SD 1,86) richtig angegeben. Vor Verlängerung des Dupilumab-Intervalls auf 4 Wochen lag die durchschnittlich richtige Identifikation bei 9,1/12 (7–12; SD 1,54). Unter den extendierten Intervallen trat keine wesentliche Veränderung, insbesondere keine Verschlechterung, auf (Abb. 3).

## Diskussion

Die Therapie mit Dupilumab als Add-on führt individuell unterschiedlich schnell zur Rückbildung der Polypen, Symptome und Komorbiditäten. Einflussfaktoren sind u. a. der Endotyp, Komorbiditäten, Ausprägung und Zeitdauer der CRSwNP und die Anzahl der durchgeführten NNH-Operationen. Eine Subgruppenanalyse der Liberty NP 52-Studie ergab ein schlechteres Ansprechen auf Dupilumab in der Patientengruppe mit multiplen FESS-Interventionen („functional endoscopic sinus surgery“) [1]. Bei den hier untersuchten 29 Patienten wurde das Dupilumab-Intervall durchschnittlich nach 13 Monaten von 300 mg/2 Wochen auf 300 mg/4 Wochen verlängert, bei 15/29 Patienten mit ≥ 3 NNH-Op. erfolgte die Intervallverlängerung erst nach durchschnittlich 17 Monaten. In der vorliegenden Untersuchung entspricht der Zeitpunkt der Intervallverlängerung nicht dem Zeitpunkt der weitestgehenden Rückbildung der Polypen und Symptome im Sinne einer kompletten Kontrolle der CRSwNP, vielmehr war bei den meisten Patienten das Intervall erst nach Bewilligung des Antrags auf Kostenübernahme bei Extension der Dupilumab-Injektionsintervalle verlängert worden. Bei einzelnen Patienten mit schwerer CRSwNP konnte unter Dupilumab als Add-on eine komplette Kontrolle bereits nach 6 Monaten erzielt werden, die meisten der Patienten der Autoren benötigten 12 Monate, einige auch länger.

### Kein Off-Label-Use

Sämtliche Patienten mit weitestgehender Rückbildung CRSwNP unter Dupilumab hatten nach einer möglichen Reduktion bzw. Beendigung der Dupilumab-Therapie gefragt. Untersuchungen zur CRSwNP nach Beendigung der Biologikatherapie, wie sie z. T. für das Asthma bronchiale existieren [5], liegen den Autoren nicht vor. Da eine Änderung der zugelassenen Dosierung als Off-Label-Use diskutiert wird [6], war vor Verlängerung der Therapieintervalle ein Antrag auf Kostenübernahme bei den entsprechenden Versicherungsträgern gestellt worden. Hiernach wurde von mehreren Gutachtern bestätigt, dass es sich bei primär gegebener Indikation für Dupilumab und anschließender Verlängerung der Applikationsintervalle infolge sehr guten Ansprechens nicht um einen Off-Label-Use handelte, zumal die Verlängerung in Anlehnung an die SINUS-52-Studie erfolgte.

### Individuelle Dosisanpassung

Im Rahmen der SINUS-52-Zulassungsstudie wurde Dupilumab 300 mg in einem Arm nach 24 Wochen nur noch 4‑wöchentlich bis Woche 52 appliziert. Unter der 4‑wöchentlichen Applikation verschlechterte sich der NPS nicht, der Nasal Congestion Score (NCS) verbesserte sich, jedoch waren die zusätzlichen Effekte hinsichtlich des NPS und des Lund-Mackay-CT-Scan-Scores im Studienarm mit fortbestehendem 2‑wöchigem Injektionsintervall größer als unter 4‑wöchigem Applikationsintervall [1]. Die zeitliche Begrenzung der SINUS-52-Studie auf 52 Wochen sowie die Verlängerung des Intervalls in einem Arm auf 4 Wochen bereits nach 24 Wochen ist sicher limitierend hinsichtlich des Therapieeffekts. Im Gegensatz zur SINUS-52-Studie wurde hier erst eine Therapieintervallverlängerung bei weitestgehender Rückbildung der CRSwNP erwogen, wenn keine systemischen Kortikosteroide eingenommen wurden und ein begleitendes Asthma bronchiale unter Kontrolle war. Die Dupilumab-Intervallverlängerung kann als explizit von den Patienten gewünschte individuelle Dosisanpassung bzw. personalisierter Therapieansatz betrachtet werden. Hierdurch können ferner das Risiko möglicher unerwünschter Nebenwirkungen und die Therapiekosten reduziert werden.

### Mögliche Ursache der Besserung

Eine Kontrollgruppe mit Beibehaltung der 2‑wöchigen Applikationsintervalle bei kompletter Kontrolle der CRSwNP wurde hier nicht mit untersucht, da es um die Frage des Erhalts der Kontrolle bei Therapieintervallverlängerung und nicht um einen zusätzlichen Nutzen durch Fortsetzung der 2‑wöchentlichen Therapie geht. Die Untersuchungen der Autoren zeigen, dass eine Verlängerung der Dupilumab-Applikationsintervalle von 2 auf 4 Wochen bzw. von 4 auf 6 Wochen bei Patienten mit kontrollierter CRSwNP nach mindestens 6 Monaten nicht zu einem Rezidiv oder einer Verschlechterung der Lebensqualität (SNOT-22, Anamnese) führt. Die Fortführung der INCS-Therapie und regelmäßige HNO-ärztliche Kontrollen sind dabei unabdingbar. Auch van der Lans et al. belegten anhand einer 2‑jährigen prospektiven Studie die hohe Effizienz von Dupilumab als Add-on bei schwerer unkontrollierter CRSwNP, eine überwiegend gute therapeutische Wirksamkeit nach 24 Wochen und die Möglichkeit, das Dupilumab-Applikationsintervall nach 24 Wochen bei der überwiegenden Zahl der Patienten ohne Verlust der CRSwNP-Kontrolle zu verlängern. Die Möglichkeit einer weiteren Intervallausdehnung nach jeweils 24 Wochen bis auf ein 8‑wöchiges Dupilumab-Intervall wurde für einige Patienten gezeigt [12]. Der anhaltenden klinischen Remission unter verlängerten Therapieintervallen liegt möglicherweise eine persistierende IL-4Rα-Sättigung mit Dupilumab infolge hoher Serumkonzentration, hoher IL-4Rα-Verfügbarkeit und möglicher Redundanz voll gesättigter Rezeptoren (IL-4Rα) zugrunde [12].

Das Therapieziel ist die Kontrolle der CRSwNP mit minimaler Behandlung, im Optimalfall eine Remission (keine Symptome, endoskopisch keine Krankheitsaktivität ≥ 12 Monate) und eine angestrebte Heilung, zusammen mit dem Bewusstsein für Nebenwirkungen [6]. Langzeitstudien mit Real-World-Evidenz zur CRSwNP unter Biologika sind unbedingt erforderlich, insbesondere besteht dringender Bedarf, für Patienten mit CRSwNP unter Kontrolle einen Zeitplan für die Biologikatherapie, eventuell ein mögliches Ende bei anhaltender Remission [6] festzulegen [5].

## Limitationen

Infolge Einschränkungen durch die Corona-Pandemie und Infektionen sowie anderen, nicht mit der CRSwNP bzw. der Therapie in Zusammenhang stehenden Ereignissen mussten Termine verschoben werden, sodass die komplette Rückbildung der Polypen und Symptome sicher häufig früher bestand. Zudem wurde die Verlängerung der Therapieintervalle durch die Anträge auf Bewilligung der Kostenübernahme weiter verzögert.

## Fazit für die Praxis

- Bei Patienten mit weitestgehender Rückbildung der chronischen Rhinosinusitis mit nasalen Polypen (CRSwNP) und der Symptome unter Dupilumab 300 mg als Add-on zu intranasalen Kortikosteroiden (INCS) ist eine Verlängerung des Dupilumab-Therapieintervalls auf 4 Wochen, danach evtl. auf 6 Wochen ohne Rezidiv möglich.
- Die Fortsetzung der INCS sowie 3‑ bis 6‑monatliche HNO-ärztliche Verlaufskontrollen sind dabei notwendig.
- Langzeitstudien mit Real-World-Evidenz zur CRSwNP unter Biologika sind erforderlich, insbesondere in Bezug darauf, für Patienten mit CRSwNP unter Kontrolle einen Zeitplan für die Biologikatherapie festzulegen.
